# Enhanced Protective Effects of Combined Treatment with *β*-Carotene and Curcumin against Hyperthermic Spermatogenic Disorders in Mice

**DOI:** 10.1155/2016/2572073

**Published:** 2016-12-05

**Authors:** Chunmei Lin, Yun Seok Choi, Seul Gi Park, Lee Wha Gwon, Jong Geol Lee, Jung-Min Yon, In-Jeoung Baek, Beom Jun Lee, Young Won Yun, Sang-Yoon Nam

**Affiliations:** ^1^College of Veterinary Medicine and Veterinary Medical Center, Chungbuk National University, Cheongju 28644, Republic of Korea; ^2^College of Chinese Medicinal Materials, Jilin Agricultural University, Changchun, Jilin 130-118, China; ^3^Asan Institute for Life Sciences, Asan Medical Center and University of Ulsan, Seoul 05505, Republic of Korea

## Abstract

Scrotal hyperthermia leads to oxidative stress and apoptosis in spermatogenic cells, which subsequently causes male infertility. In this study, we examined the effects of *β*-carotene and/or curcumin on heat-stress- (HS-) induced testicular injuries in mice. ICR male mice (8 weeks old) were consecutively treated with *β*-carotene (10 mg/kg) and/or curcumin (20 mg/kg) orally once a day for 14 days and then subjected to single exposure with scrotal HS at 43°C for 15 min on day 7. HS induced a significant reduction in testicular weight, appearance of multinucleated giant cells, and desquamation of germ cells in destructive seminiferous tubules, as well as degenerative Leydig cells. Moreover, HS reduced the superoxide dismutase (SOD) activity and mRNA levels of mitochondrial SOD, phospholipid hydroperoxide glutathione peroxidase, B-cell lymphoma-extra-large, and 3*β*-hydroxysteroid dehydrogenase, with increases in lipid peroxidation levels and mRNA levels of BCL2-associated X protein and caspase-3 relative to those of the control group. However, these changes were significantly recovered by combined treatment with *β*-carotene and curcumin after HS. These findings indicate that the combined treatment with *β*-carotene and curcumin might be a valuable protective agent to ameliorate hyperthermic spermatogenic disorders via its potent antioxidative, antiapoptotic, and androgen synthetic effects.

## 1. Introduction

In most mammals, testes must be maintained at 2–8°C lower than body temperature for normal spermatogenesis [1, 2]. However, modern lifestyles can induce scrotal hyperthermia, resulting in male infertility. Indeed, testicular heating caused by taking a sauna or hot bath, close-fitting underwear, using a laptop closely to legs, driving, and electric blankets could be detrimental to spermatogenesis [3]. Scrotal heat stress (HS) induces severe oxidative stress in mouse testes, eventually causing germ cell death [4]. Generation of reactive oxygen species (ROS) such as superoxide appears to be involved in these effects [5]. Apoptosis was noted most frequently among primary spermatocytes and round spermatids in the primary germ cell stage following HS [6]. The germ cell stages most sensitive to local HS are the premeiotic tetraploid leptotene, zygotene, and pachytene during formation of primary spermatocytes and are the first step of spermatids following the second meiotic division [7, 8]. Vacuolation of the germinal epithelium and formation of multinucleated giant cells were observed after the onset of germ cell loss by scrotal HS [6]. Spermatogenic cell apoptosis caused by heat-induced oxidative stress is a potential mechanism leading to impaired spermatogenesis.

Carotenoids are natural pigments that have long been used for medical, cosmetic, and dietary purposes [9]. Major sources of dietary *β*-carotene include green leafy vegetables, as well as orange and yellow fruits and vegetables [10]. Carotenoids such as lycopene and *β*-carotene are important biological compounds that can participate in free radical reactions, finally contributing to the antioxidant defense system [11]. Beta-carotene and related carotenoids have been shown to decrease the rate of formation of methyl linoleate hydroperoxides [12], while curcumin, a yellow-orange polyphenol present in the rhizome of turmeric (*Curcuma longa* L., Zingiberaceae), is known to have a wide array of pharmacological and biological activities [13]. Curcumin has been reported to have preventive or putative therapeutic activities owing to its antioxidant [14], anti-inflammatory [13, 15], antidepressant [16], antimicrobial [17], antimutagenic [18], and anti-HIV properties [19]. Inhibition of peroxidation by curcumin is primarily attributed to the scavenging of reactive free radicals involved in peroxidation [20].

Therefore, this study was conducted to investigate the effects of *β*-carotene and/or curcumin against HS-induced spermatogenic disorders as part of the search for an effective strategy for treatment of male infertility.

## 2. Materials and Methods

### 2.1. Animals and Chemicals

ICR male mice (8 weeks old; Orient Bio, Gyeonggi, Korea) were housed in polycarbonate cages in a well-ventilated room maintained at 21 ± 2°C and 55 ± 10% relative humidity under a 12-hour light/dark cycle. Mice were fed standard mouse chow (Samyang Ltd., Incheon, Korea) and tap water ad libitum. All experiments were approved by the Chungbuk National University Animal Care Committee and carried out according to the* Guide for the Care and Use of Laboratory Animals* (Chungbuk National University Animal Care Committee, CBNUA-469-13-02).

*β*-Carotene, curcumin, and olive oil were purchased from Sigma Chemical Company (St. Louis, MO, USA) and Junsei Chemical Company (Tokyo, Japan).

### 2.2. Experimental Design

The mice were randomly divided into the normal control group treated with vehicle (Con), heat stress (HS) group, HS plus *β*-carotene group (HS + *β*-CA), HS plus curcumin administration group (HS + Cur), and HS plus *β*-carotene and curcumin administration group (HS + *β*-CA + Cur). The best effective doses of *β*-carotene and curcumin were determined to be 3.4 mg/mL (10 mg/kg body weight) and 6.7 mg/mL (20 mg/kg body weight) in vehicle (olive oil), respectively, based on our preliminary experiments. All substances were administered via intragastric intubation once a day for 14 consecutive days. Seven days after treatment, mice were subjected to a single HS under intramuscular anesthesia with Zoletil at 10 *μ*L/10 g body weight (Virbac, Carros, France). During heat exposure, the lower third of the body, including the scrotum, tail, and hind legs, was submerged in a 43°C water bath for 15 min. After being returned to their cages, the animals were placed on a warm mat (25°C) to maintain their body temperature until they had fully recovered from the anesthesia. At 7 days after heat exposure, all animals were sacrificed under diethyl ether anesthesia, after which the testes were collected and stored at −80°C until further use for biochemical and histopathological investigations.

### 2.3. Measurement of Body and Testis Weights

The body weights were measured daily during the experimental periods and the weight of each testis was recorded on day 14 immediately after sacrifice.

### 2.4. Histopathology

Testes were fixed in Bouin's solution and processed according to routine histological techniques. After paraffin embedment, 4 *μ*m sections were stained with hematoxylin and eosin for histopathologic evaluation.

### 2.5. Lipid Peroxidation Measurement

Lipid peroxidation levels in the testes were measured using the thiobarbituric-acid-reactive substances (TBARS) method. The TBARS concentration in the testes was measured spectrophotometrically and expressed as the malondialdehyde (MDA) level. The concentration of TBARS was expressed as nmol MDA/mg protein using tetramethoxypropane (TMP) as a standard. Testis tissues were homogenized in 1.8 mL of cold 10 mM phosphate buffer, after which 1 mL of the homogenate was mixed with 1 mL of 8.1% SDS solution and 2 mL of 20% acetic acid solution. After adding 1 mL of 0.75% TBA solution to this mixture, it was heated for 30 min in a 95°C oven. The mixture was then cooled at room temperature and centrifuged at 4220 ×g for 15 min, after which the absorbance of the supernatant was measured at 532 nm using a spectrophotometer. The value was subsequently determined based on comparison to a TMP standard curve. Protein concentrations were measured using bovine serum albumin as a standard.

### 2.6. Superoxide Dismutase (SOD) Activity Assay

Total SOD activity was assayed using an SOD Assay Kit-WST (Dojindo Laboratories, Kumamoto, Japan). Briefly, testis tissues were homogenized and the protein concentrations of the supernatant were analyzed. The supernatants were then incubated with an assay reagent containing xanthine, xanthine oxidase, and a water-soluble tetrazolium salt (WST-1). The superoxide free radicals generated from the xanthine by xanthine oxidase reduced WST-1 to WST-1 diformazan, which shows its maximum absorbance at 450 nm. SOD in the testes inhibits the WST-1 reduction since the enzyme catalyzes the dismutation of superoxide ions to molecular oxygen and hydrogen peroxide. The reduction of WST-1 was measured spectrophotometrically at 450 nm and SOD activity was calculated as an inhibition rate in which 1 U was defined as a 50% decrease from the control value over a period of 30 min at 37°C. The results were presented as specific activity calculated as the total SOD activity per testis divided by the total amount of protein per testis. Data from five independent experiments were analyzed.

### 2.7. Real-Time PCR Analysis

Total RNA was isolated from mouse testes using TRIzol Reagent (Invitrogen, CA, USA) according to the manufacturer's protocol. Total RNA concentrations were determined by UV absorbance, after which 2 *μ*g of total RNA was reverse-transcribed using random primers and High Capacity cDNA Reverse Transcription Kits (Applied Biosystems, Foster City, CA, USA). Quantitative PCR was performed using the SYBR Green Master Mix (Applied Biosystems) and testes cDNA (1.6 *μ*g) as a template. Reactions were performed using a 7500 Real-Time PCR System (Applied Biosystems) according to the manufacturer's instructions. Gene-specific primers were designed by TIB Mol-Bio Synthesis (Berlin, Germany). Mouse primers specific for genes encoding the antioxidant enzymes mitochondrial SOD (SOD2) and phospholipid hydroperoxide glutathione peroxidase (PHGPx), as well as the apoptosis-related genes Bax, Bcl-xL, and caspase-3, and those encoding the androgen synthesis enzyme 3*β*-hydroxysteroid dehydrogenase (3*β*-HSD) in Leydig cells were utilized. Expression was normalized against GAPDH as an internal standard to determine the target transcript expression. The primer sequences used in this study were as follows: SOD2 (NM_013671), forward 5′-GGAGCAAGGTCGCTTACAGA-3′ and reverse 5′-GTGCTCCCACACGTCAATC-3′; PHGPx (NM_008162), forward 5′-TAAGAACGGCTGCGTGGT-3′ and reverse 5′-GTAGGGGCACACACTTGTAGG-3′; caspase-3 (NM_009810), forward 5′-AAAGCCGAAACTCTTCATCAT-3′ and reverse 5′-GTCCCACTGTCTTCA-3′; Bcl-xL, forward 5′-TGACCACCTAGAGCCTTGGA-3′ and reverse 5′-TGTTCCCGTAGAGATCCACAA-3′; Bax (NM_0075273), forward 5′-CTCAAGGCCCTGTGCACTAA-3′ and reverse 5′-CACGGAGGAAGTCCAGTGTC-3′; 3*β*-HSD (NM_153193), forward 5′-GACTGCTGACACACCACACC-3′ and reverse 5′-GGGAGTGAGGTTAACTTAATGTACG-3′; GAPDH (NM_008084), forward 5′-CGTGCCGCCTGGAGAAACC-3′ and reverse 5′-TGGAAGAGTGGGAGTTGCTGTTG-3′. Each PCR program was started with UNG (uracil-N-glycosylase) incubation at 50°C for 2 min, followed by incubation at 95°C for 10 min. This was followed by 40 cycles of denaturation at 95°C for 15 sec and annealing and extension at 60°C for 1 min. Data were acquired and analyzed with the 7500 system SDS software (version 1.3.1.21, Applied Biosystems). Amplification kinetics were recorded in real-time mode as sigmoid process curves for which fluorescence was plotted against the number of amplification cycles. Data from five independent runs were analyzed using a comparative Ct method.

### 2.8. Statistical Analysis

Differences among groups were assessed by one-way analysis of variance (ANOVA) followed by Tukey's multiple comparison test. *p* values <0.05 were considered statistically significant. All data were expressed as the mean ± SEM. All analyses were conducted using SPSS for Windows software, version 10.0 (SPSS Inc., Chicago, IL, USA).

## 3. Results

### 3.1. Testicular Weight Changes

**Figure 1:**
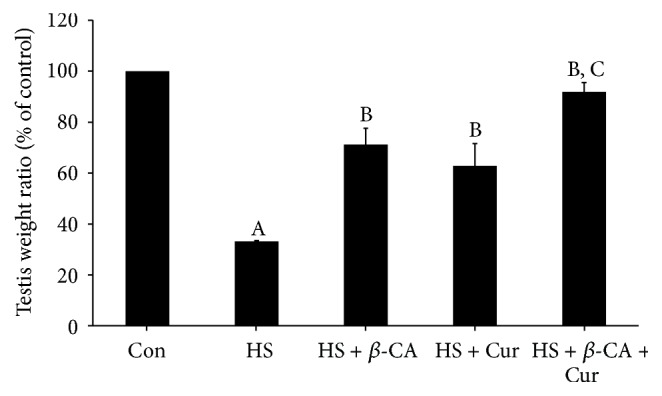
Relative testis weight (% of control) following treatment with *β*-carotene (*β*-CA) and/or curcumin (Cur) after heat stress (HS) in mice. Results are the means ± SEM (*n* = 10). Significant difference between each treatment group versus normal control (Con; A), HS (B), or HS + Cur (C) group as evaluated by one-way ANOVA at *p* < 0.05.

Figure 1 shows the testicular weights of treated animals relative to the normal control group. The testicular weight ratio of the HS group (33.2%) was significantly lower than that of the control group (100%; *p* < 0.05). However, the testicular weight ratios in the *β*-carotene or curcumin cotreatment groups with HS were significantly higher than that of the HS group (71.2% or 67.5%; *p* < 0.05, resp.). The testis weight ratio in the combined *β*-carotene and curcumin group under HS was restored to almost normal (91.9%; *p* < 0.05). In addition, there were no significant differences in body weights among all groups during the experimental period (data not shown).

### 3.2. Spermatogenic Activity

**Figure 2:**
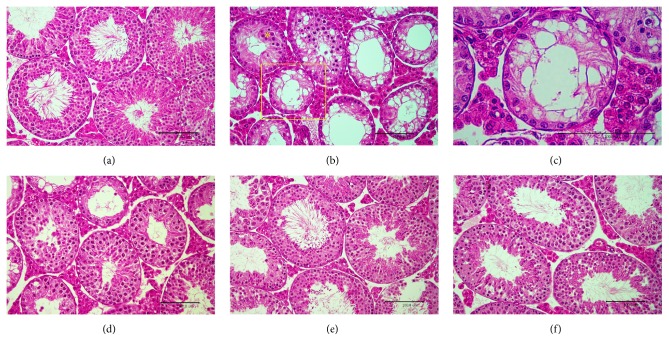
Representative histopathological findings of testes treated with *β*-carotene (*β*-CA) and/or curcumin (Cur) after heat stress (HS) in mice. (a) Normal control group. (b) HS with severe testicular degeneration such as abnormal spermatocytes and spermatids, vacuolation of seminiferous tubules, formation of multinucleated giant cells (yellow asterisk), and hyperplasia of Leydig cells. (c) Magnified area of (b) (yellow box). (d) HS plus *β*-CA group. (e) HS plus Cur group. (f) HS plus *β*-CA + Cur group. H&E staining; bar: 100 *μ*m.

Exposure of testes to exogenous hyperthermia caused severe damage in the seminiferous tubules when compared with normal control testis (Figure 2(a)), resulting in much smaller diameters than the normal control group. A large proportion of seminiferous tubules in the atrophic testes showed signs of degeneration, vacuolation, and disorganization (Figure 2(b)). Formation of multinucleated giant cells, destructive spermatocytes and spermatids, and hyperplasia of Leydig cells characterized the extent of injury (Figure 2(c)). However, mice subjected to HS in conjunction with *β*-carotene or curcumin treatment showed significant improvement in spermatogenic activity relative to the HS group (Figures 2(d) and 2(e)). This was especially true in the testes of mice treated with combined *β*-carotene and curcumin, for which structure was restored almost to that of normal testes, tubular morphology was preserved, and many tubules had abundant spermatocytes and spermatids (Figure 2(f)).

### 3.3. Lipid Peroxidation Level

**Figure 3:**
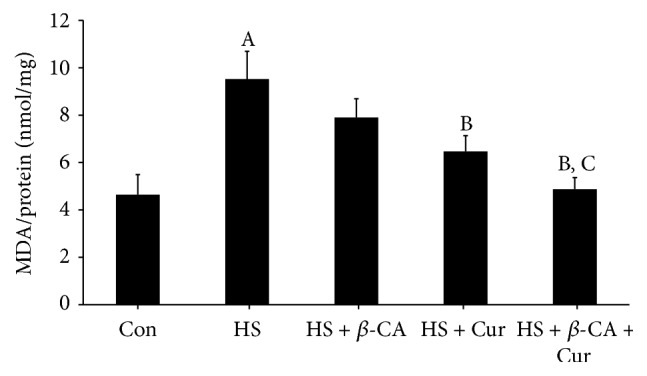
Protective effects of *β*-carotene (*β*-CA) and/or curcumin (Cur) against oxidative damage induced by heat stress (HS) in mouse testes. Lipid peroxidation was determined by thiobarbituric-acid-reactive species levels and expressed as the malondialdehyde (MDA) level. Results are the means ± SEM (*n* = 10). Significant difference between each treatment group versus normal control (Con; A), HS (B), or HS + Cur (C) as evaluated by one-way ANOVA at *p* < 0.05.

According to Figure 3, mouse testes exposed to HS alone exhibited a significant increase in MDA level (9.52 ± 1.17 nmol/mg) compared to the control group (3.93 ± 0.85 nmol/mg; *p* < 0.05). However, MDA levels in the *β*-carotene or curcumin cotreatment group after HS decreased relative to the control group (7.90 ± 0.79 nmol/mg or 6.46 ± 0.67 nmol/mg; *p* < 0.05, resp.). Particularly, the MDA level in mice that received combined treatment with *β*-carotene and curcumin after HS was reduced to almost the control levels (4.87 ± 0.50 nmol/mg; *p* < 0.05).

### 3.4. SOD Activity Level

**Figure 4:**
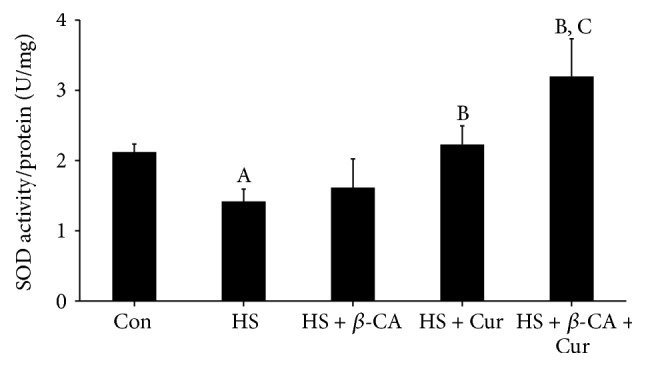
Superoxide dismutase (SOD) activity levels in mouse testes induced by heat stress (HS) in the *β*-carotene (*β*-CA) and/or curcumin (Cur) treated groups. Results are the means ± SEM (*n* = 10). Significant differences between each treatment group and normal control (Con; A), HS (B), or HS + Cur (C) group were examined at *p* < 0.05.

According to Figure 4, mouse testes exposed to a transient HS alone exhibited significantly reduced SOD activity (1.42 ± 0.18 U/mg) compared to the control group (2.12 ± 0.12 U/mg; *p* < 0.05). However, the SOD activity of the *β*-carotene or curcumin treatment group with HS was greatly increased (1.65 ± 0.27 or 2.09 ± 0.26 U/mg, resp.; *p* < 0.05). Furthermore, the SOD activity in mice that received the combined treatment with *β*-carotene and curcumin after HS was significantly increased relative to both single treatment groups (3.20 ± 0.36 nmol/mg; *p* < 0.05).

### 3.5. Gene Expression Patterns of Antioxidant Enzymes

**Figure 5:**
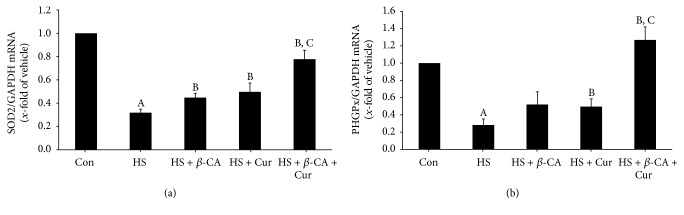
Quantitative real-time PCR analysis of antioxidant enzyme mRNA levels in mouse testes induced by heat stress (HS) in the *β*-carotene (*β*-CA) and/or curcumin (Cur) treated groups. (a) Mitochondrial manganese superoxide dismutase (SOD2). (b) Phospholipid hydroperoxide glutathione peroxidase (PHGPx). Results are the means ± SEM (*n* = 10). Significant differences between each treatment group and normal control (Con; A), HS (B), or HS + Cur (C) group were examined at *p* < 0.05. GAPDH was used as an internal standard to normalize target transcript expressions.

Superoxide dismutase 2 mRNA level in mouse testes exposed to a transient HS was 0.32 times that of the control group (1-fold; *p* < 0.05). However, when mice were cotreated with *β*-carotene or curcumin after HS, the SOD2 mRNA level was 0.45 times or 0.50 times that of the control, which was greater than that of the HS group. Furthermore, the SOD2 mRNA level of the group treated with combined *β*-carotene and curcumin after HS was 0.78 times that of the control and significantly greater than that of the remaining groups (*p* < 0.05; Figure 5(a)).

PHGPx mRNA level in mouse testes exposed to a transient HS was 0.28 times that of the control group (1-fold; *p* < 0.05). However, when mice were cotreated with *β*-carotene or curcumin after HS, the PHGPx mRNA level was 0.52 times or 0.50 times that of the control, respectively, and greater than that of the HS group. Particularly, the PHGPx mRNA level in the group treated with combined *β*-carotene and curcumin after HS was 1.27 times that of the control, which was significantly greater than that of the remaining groups (*p* < 0.05; Figure 5(b)).

### 3.6. Gene Expression Patterns of Apoptosis-Related Agents

**Figure 6:**
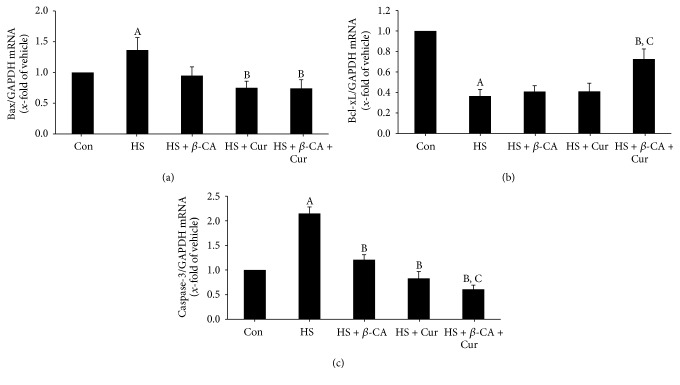
Quantitative real-time PCR analysis of apoptosis-related enzyme mRNA levels in mouse testis induced by heat stress (HS) in the *β*-carotene (*β*-CA) and/or curcumin (Cur) treated groups. (a) Bax. (b) Bcl-xL. (c) Caspase-3. Results are the means ± SEM (*n* = 10). Significant differences between each treatment group and normal control (Con; A), HS (B), or HS + Cur (C) group were examined at *p* < 0.05. GAPDH was used as an internal standard to normalize target transcript expressions.

Bax mRNA level in mouse testes exposed to a transient HS was 1.36 times that of the control group (1-fold; *p* < 0.05). However, when mice were cotreated with *β*-carotene or curcumin after HS, Bax mRNA levels were 0.95 times and 0.75 times that of the control, respectively, which was lower than that of the HS group. Furthermore, the Bax mRNA level in the group treated with combined *β*-carotene and curcumin after HS was significantly reduced to 0.74 times that of the control relative to the HS group (*p* < 0.05; Figure 6(a)).

Bcl-xL mRNA level in the testes of mice exposed to a transient HS was 0.37 times that of the control group (1-fold; *p* < 0.05). When mice were cotreated with *β*-carotene or curcumin after HS, the Bcl-xL mRNA level was 0.41 times or 0.42 times that of the control, respectively, similar to that of the HS group. However, the Bcl-xL mRNA level in the group treated with combined *β*-carotene and curcumin after HS was 0.73 times that of the control, which was significantly higher than those of other heat-exposed groups (*p* < 0.05; Figure 6(b)).

Caspase-3 mRNA levels in mouse testes exposed to a transient HS were 2.15 times higher than that of the control group (1-fold; *p* < 0.05). However, when mice were cotreated with *β*-carotene or curcumin after HS, the caspase-3 mRNA level was 1.21 times or 0.83 times lower than that of the HS group. Furthermore, the caspase-3 mRNA level in the group treated with combined *β*-carotene and curcumin after HS was 0.61 times that of the control and significantly lower than those of other heat-exposed groups (*p* < 0.05; Figure 6(c)).

### 3.7. Gene Expression Patterns of 3*β*-HSD

**Figure 7:**
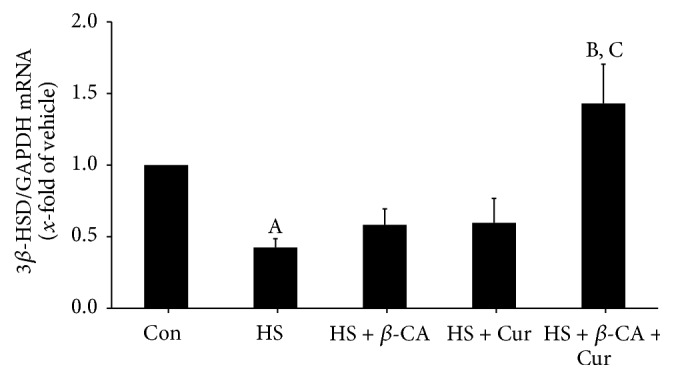
Quantitative real-time PCR analysis of 3*β*-HSD mRNA levels in mouse testis induced by heat stress (HS) in the *β*-carotene (*β*-CA) and/or curcumin (Cur) treated groups. Results are the means ± SEM (*n* = 10). Significant differences between each treatment group and normal control (Con; A), HS (B), or HS + Cur (C) group were examined at *p* < 0.05. GAPDH was used as an internal standard to normalize target transcript expression.

3*β*-HSD mRNA levels in the testes of mice exposed to a transient HS were 0.42 times that of the control group (1-fold; *p* < 0.05). When mice were treated with *β*-carotene or curcumin after HS, the 3*β*-HSD mRNA level (0.58 times or 0.60 times that of the control, resp.) was slightly increased relative to the HS group. However, the 3*β*-HSD mRNA level in the group treated with combined *β*-carotene and curcumin after HS was 1.43 times that of the control and significantly higher than those of other heat-exposed groups (*p* < 0.05; Figure 7).

## 4. Discussion

Many studies have demonstrated reproductive toxicity due to testicular hyperthermia in mammals. Testicular function is temperature-dependent and testicular hyperthermia can induce several clinical problems, including infertility [4]. Therefore, it is useful for our healthy life to identify safe preventive and therapeutic agents from dietary natural materials for treatment of HS-induced testicular dysfunction. *β*-Carotene has been reported to inactivate harmful free radicals produced by various stressors [21], including antiapoptotic and antioxidant effects on methotrexate-induced testicular injury in rats [22] and ethanol-induced hepatic cell death by inhibiting caspase-9 and caspase-3 expression [23]. Previous studies have demonstrated that curcumin plays preventive or putative therapeutic roles by acting as an antioxidant [14] and phenolic groups in curcumin eliminate oxygen-derived free radicals and superoxide anions [24]. Recently, we demonstrated that curcumin dose-dependently improves spermatogenic disorders induced by scrotal heat stress in mice [25]. Therefore, *β*-carotene and curcumin could be good natural protective candidates to protect against male infertility induced by various environmental stressors. In the present study, we confirmed the enhanced effects of combined treatment with *β*-carotene and curcumin against HS-induced testicular damage in mice.

Scrotal hyperthermia disrupted spermatogenesis and eventually caused germ cell death [4]. In the present study, scrotal HS (43°C, 15 min) led to decreased testis weight relative to that of control mice. However, the reduced testicular weights in HS mice were recovered by cotreatment with *β*-carotene or curcumin, with the weight in the group treated with combined *β*-carotene and curcumin recovering to almost that of the control. Primary spermatocytes and round spermatids were most sensitive to HS [6], while spermatogonia showed resistance to heat [26]. Consistent with these results, our study showed that HS induced destructive spermatocytes and spermatids within degenerative seminiferous tubules, as well as sloughing, atrophy, vacuolation, and apoptosis of germ cells. However, cotreatment with *β*-carotene or curcumin led to repair activity, as indicated by the presence of many spermatogenic cells. In particular, the combined treatment with *β*-carotene and curcumin resulted in recovery to almost normal testicular morphology.

Scrotal HS, which is one of the major causes of impaired spermatogenesis, is deeply associated with oxidative stress and apoptosis of germ cells [27]. In the present study, scrotal HS increased the lipid peroxidation level and decreased the SOD activity in testes. A previous study revealed that *β*-carotene contributes to the antioxidant defense system by participating in free radical scavenging reactions [11] and that curcumin has the ability to inhibit the generation of ROS by preventing oxidation in rat peritoneal macrophages [24]. The results of our study also showed that *β*-carotene and/or curcumin treatment groups with HS recovered these oxidative stress responses and damaged antioxidant enzyme levels to the control levels by ROS scavenging. Moreover, we found that the combined treatment with *β*-carotene and curcumin remarkably reversed the testicular dysfunction following HS relative to the individual treatment groups.

SOD and GSH-dependent enzymes including PHGPx are key enzymes of cellular resistance to oxidative stress that are crucial to understanding testicular antioxidant defense mechanisms [28]. Cryptorchidism is associated with high levels of lipid peroxidation and reduction in PHGPx activity in the testes [29]. In the present study, the HS group showed considerably reduced levels of SOD2 and PHGPx, but the *β*-carotene or curcumin treatment group with HS showed slightly recovered antioxidant enzyme mRNA expression. In particular, the levels in groups that received the combined treatment were significantly restored relative to the *β*-carotene or curcumin alone group. These results indicate that the combination of *β*-carotene and curcumin could enhance antioxidative potential against HS via activation of the representative antioxidative enzymes in testes.

Local heating of the scrotum at 43°C for 15 min can induce germ cells to undergo apoptosis [26]. After the onset of cellular stress, proapoptotic (Bax) and antiapoptotic (Bcl-2) proteins exist in key regulators of apoptosis [30]. Increasing levels of Bax expression accelerate cell death, and the increased Bcl-2 improves cell survival [31]. Beta-carotene prevented ethanol-induced hepatic cell death by inhibiting caspase-9 and caspase-3 expressions [23]. In the present study, Bax and caspase-3 mRNA levels increased considerably, but Bcl-xL mRNA expression decreased remarkably in the testes of the HS group. However, the apoptotic damage induced by exogenous heat exposure was prevented by cotreatment with *β*-carotene or curcumin. Furthermore, the combined treatment with both natural products after HS recovered the expression of apoptotic genes related to cell death to almost normal relative to the *β*-carotene or curcumin alone treatment. These results indicate that the combined treatment with *β*-carotene and curcumin could strongly protect spermatogenic cells against HS-induced apoptosis via the enhancement of their antiapoptotic activities.

Scrotal hyperthermia damaged Leydig cell function and eventually inhibited the synthesis of testosterone in rats [32]. In the adrenal glands, testes, and ovaries, 3*β*-HSD plays a key role in testosterone synthesis [33]. In the present study, 3*β*-HSD mRNA levels were considerably lower in the testes of the HS group, but the combined treatment with *β*-carotene and curcumin after HS significantly increased the 3*β*-HSD mRNA level relative to the other heat-exposed groups as well as *β*-carotene or curcumin alone group. These results suggest that combination of *β*-carotene and curcumin could restore testosterone biosynthesis following HS via modulation of the 3*β*-HSD gene.

## 5. Conclusions

In summary, the results of this study indicate that combined treatment with *β*-carotene and curcumin might be a valuable protective agent to ameliorate HS-induced male infertility through the potent antioxidative, antiapoptotic, and androgen synthesis effects.

## References

[1] Hughes I. A., Acerini C. L. (2008). Factors controlling testis descent. *European Journal of Endocrinology*.

[2] Vendramini V., Sasso-Cerri E., Miraglia S. M. (2010). Amifostine reduces the seminiferous epithelium damage in doxorubicin-treated prepubertal rats without improving the fertility status. *Reproductive Biology and Endocrinology*.

[3] Ivell R. (2007). Lifestyle impact and the biology of the human scrotum. *Reproductive Biology and Endocrinology*.

[4] Paul C., Teng S., Saunders P. T. K. (2009). A single, mild, transient scrotal heat stress causes hypoxia and oxidative stress in mouse testes, which induces germ cell death. *Biology of Reproduction*.

[5] Romeo C., Ientile R., Impellizzeri P. (2003). Preliminary report on nitric oxide-mediated oxidative damage in adolescent varicocele. *Human Reproduction*.

[6] Henriksen K., Hakovirta H., Parvinen M. (1995). In-situ quantification of stage-specific apoptosis in the rat seminiferous epithelium: effects of short-term experimental cryptorchidism. *International Journal of Andrology*.

[7] Blackshaw A. W., Hamilton D., Massey P. F. (1973). Effect of scrotal heating on testicular enzymes and spermatogenesis in the rat. *Australian Journal of Biological Sciences*.

[8] Chowdhury A. K., Steinberger E. (1970). Early changes in the germinal epithelium of rat testes following exposure to heat. *Journal of Reproduction and Fertility*.

[9] Krinsky N. I. (1989). Carotenoids as chemopreventive agents. *Preventive Medicine*.

[10] Krinsky N. I., Johnson E. J. (2005). Carotenoid actions and their relation to health and disease. *Molecular Aspects of Medicine*.

[11] Di Mascio P., Murphy M. E., Sies H. (1991). Antioxidant defense systems: the role of carotenoids, tocopherols, and thiols. *American Journal of Clinical Nutrition*.

[12] Terao J. (1989). Antioxidant activity of *β*-carotene-related carotenoids in solution. *Lipids*.

[13] Araújo C. A. C., Leon L. L. (2001). Biological activities of *Curcuma longa* L.. *Memorias do Instituto Oswaldo Cruz*.

[14] Piper J. T., Singhal S. S., Salameh M. S., Torman R. T., Awasthi Y. C., Awasthi S. (1998). Mechanisms of anticarcinogenic properties of curcumin: the effect of curcumin on glutathione linked detoxification enzymes in rat liver. *The International Journal of Biochemistry & Cell Biology*.

[15] Sharma S., Chopra K., Kulkarni S. K. (2007). Effect of insulin and its combination with resveratrol or curcumin in attenuation of diabetic neuropathic pain: participation of nitric oxide and TNF-alpha. *Phytotherapy Research*.

[16] Xu Y., Ku B.-S., Yao H.-Y. (2005). Antidepressant effects of curcumin in the forced swim test and olfactory bulbectomy models of depression in rats. *Pharmacology Biochemistry and Behavior*.

[17] Negi P. S., Jayaprakasha G. K., Jagan Mohan Rao L., Sakariah K. K. (1999). Antibacterial activity of turmeric oil: a byproduct from curcumin manufacture. *Journal of Agricultural and Food Chemistry*.

[18] Nagabhushan M., Amonkar A. J., Bhide S. V. (1987). In vitro antimutagenicity of curcumin against environmental mutagens. *Food and Chemical Toxicology*.

[19] Jordan W. C., Drew C. R. (1996). Curcumin—a natural herb with anti-HIV activity. *Journal of the National Medical Association*.

[20] Ak T., Gülçin I. (2008). Antioxidant and radical scavenging properties of curcumin. *Chemico-Biological Interactions*.

[21] El-Demerdash F. M., Yousef M. I., Kedwany F. S., Baghdadi H. H. (2004). Cadmium-induced changes in lipid peroxidation, blood hematology, biochemical parameters and semen quality of male rats: protective role of vitamin E and *β*-carotene. *Food and Chemical Toxicology*.

[22] Vardi N., Parlakpinar H., Ates B., Cetin A., Otlu A. (2009). Antiapoptotic and antioxidant effects of *β*-carotene against methotrexate-induced testicular injury. *Fertility and Sterility*.

[23] Peng H.-C., Chen J.-R., Chen Y.-L., Yang S.-C., Yang S.-S. (2010). *β*-Carotene exhibits antioxidant and anti-apoptotic properties to prevent ethanol-induced cytotoxicity in isolated rat hepatocytes. *Phytotherapy Research*.

[24] Reddy A. C. P., Lokesh B. R. (1994). Studies on the inhibitory effects of curcumin and eugenol on the formation of reactive oxygen species and the oxidation of ferrous iron. *Molecular and Cellular Biochemistry*.

[25] Lin C., Shin D.-G., Park S. G. (2015). Curcumin dose-dependently improves spermatogenic disorders induced by scrotal heat stress in mice. *Food & Function*.

[26] Yin Y., Hawkins K. L., DeWolf W. C., Morgentaler A. (1997). Heat stress causes testicular germ cell apoptosis in adult mice. *Journal of Andrology*.

[27] Shiraishi K., Takihara H., Matsuyama H. (2010). Elevated scrotal temperature, but not varicocele grade, reflects testicular oxidative stress-mediated apoptosis. *World Journal of Urology*.

[28] Bauché F., Fouchard M.-H., Jégou B. (1994). Antioxidant system in rat testicular cells. *FEBS Letters*.

[29] Jung K. Y., Yon J.-M., Lin C. (2015). Phospholipid hydroperoxide glutathione peroxidase is involved in the maintenance of male fertility under cryptorchidism in mice. *Reproductive Toxicology*.

[30] Çeribaşı A. O., Sakin F., Türk G., Sönmez M., Ateşşahin A. (2012). Impact of ellagic acid on adriamycin-induced testicular histopathological lesions, apoptosis, lipid peroxidation and sperm damages. *Experimental and Toxicologic Pathology*.

[31] Oltval Z. N., Milliman C. L., Korsmeyer S. J. (1993). Bcl-2 heterodimerizes in vivo with a conserved homolog, Bax, that accelerates programed cell death. *Cell*.

[32] Aktas C., Kanter M. (2009). A morphological study on Leydig cells of scrotal hyperthermia applied rats in short-term. *Journal of Molecular Histology*.

[33] Sholl S. A. (1983). 3*β*-Hydroxysteroid *dehydrogenase/Δ^5-4^ isomerase* activity in the rhesus monkey placenta and fetal adrenal, testis and ovary during late gestation. *Steroids*.

